# Evolution of the Retroviral Restriction Gene *Fv1*: Inhibition of Non-MLV Retroviruses

**DOI:** 10.1371/journal.ppat.1003968

**Published:** 2014-03-06

**Authors:** Melvyn W. Yap, Emily Colbeck, Scott A. Ellis, Jonathan P. Stoye

**Affiliations:** 1 Division of Virology, National Institute for Medical Research, Mill Hill, London, United Kingdom; 2 Faculty of Medicine, Imperial College London, London, United Kingdom; University of Massachusetts Medical School, United States of America

## Abstract

Fv1 is the prototypic restriction factor that protects against infection by the murine leukemia virus (MLV). It was first identified in cells that were derived from laboratory mice and was found to be homologous to the *gag* gene of an endogenous retrovirus (ERV). To understand the evolution of the host restriction gene from its retroviral origins, *Fv1*s from wild mice were isolated and characterized. Most of these possess intact open reading frames but not all restricted N-, B-, NR-or NB-tropic MLVs, suggesting that other viruses could have played a role in the selection of the gene. The Fv1s from *Mus spretus* and *Mus caroli* were found to restrict equine infectious anemia virus (EIAV) and feline foamy virus (FFV) respectively, indicating that Fv1 could have a broader target range than previously thought, including activity against lentiviruses and spumaviruses. Analyses of the *Fv1* sequences revealed a number of residues in the C-terminal region that had evolved under positive selection. Four of these selected residues were found to be involved in the novel restriction by mapping studies. These results strengthen the similarities between the two capsid binding restriction factors, *Fv1* and TRIM5α, which support the hypothesis that *Fv1* defended mice against waves of retroviral infection possibly including non-MLVs as well as MLVs.

## Author Summary

We have followed the evolution of the retroviral restriction gene, *Fv1*, by functional analysis. We show that *Fv1* can recognize and restrict a wider range of retroviruses than previously thought including examples from the gammaretrovirus, lentivirus and foamy virus genera. Nearly every *Fv1* tested showed a different pattern of restriction activity. We also identify several hypervariable regions in the coding sequence containing positively selected amino acids that we show to be directly involved in determining restriction specificity. Our results strengthen the analogy between *Fv1* and another capsid-binding, retrovirus restriction factor, TRIM5α. Although they share no sequence identity they appear to share a similar design and appear likely to recognise different targets by a mechanism involving multiple weak interactions between a virus-binding domain containing several variable regions and the surface of the viral capsid. We also describe a pattern of constant genetic change, implying that different species of *Mus* have evolved in the face of ever-changing retroviral threats by viruses of different kinds.

## Introduction

Viruses co-evolve with their hosts, upon which they are completely dependent for replication. As the host acquires strategies to restrict virus infection the invaders develop counter measures to evade restriction. The ensuing genetic conflict can play out over an extensive timeframe [1], [2], [3], [4]. Due to the unique replication strategy employed by retroviruses where integration of viral genetic information into the host genome occurs [5], the conflict between virus and host can take an interesting twist. When integration occurs in germ or embryonic cells, the virus can become an endogenous retrovirus (ERV) and inherited through the germ line [6], [7]. As a result, viral gene products can be conscripted to serve as defensive forces against further viral infection [8]. The murine retrovirus restriction gene, *Fv1*, provides perhaps the prototypic example of one such gene [9].

Fv1 restriction was first described in the early 1970s [10], [11] as an activity protecting mice against infection with murine leukemia virus (MLV). Two semi-dominant alleles were identified, *Fv1^n^* and *Fv1^b^*, that provide protection against B-tropic and N-tropic MLVs, respectively [12], [13]. The crucial difference between N-tropic and B-tropic MLV maps within the viral *gag* gene to a single codon encoding amino acid 110 of the mature capsid (CA) protein [14] indicating that CA represents the target for the restriction factor. MLVs insensitive to *Fv1*, called NB-tropic, carry further changes in CA [15], [16]. The mode of action of the Fv1 protein is not fully understood but indirect evidence suggests that it binds to CA on the cores of incoming virions shortly after virus entry into the cell without inhibiting viral reverse transcription [17] but somehow preventing entry of newly synthesized viral DNA into the nucleus [9]. Based on sequence similarity, *Fv1* appears to be derived from the *gag* gene of an ancient ERV called MERV-L (murine endogenous retrovirus with a leucine tRNA primer binding site) though it appears only distantly related to MLV [18].

Amino acid 110 of CA also determines sensitivity of MLV to another retrovirus restriction factor, TRIM5α [19], best known for its ability to restrict HIV-1 [20]. While there is no similarity between Fv1 and TRIM5α at the primary sequence level, both molecules share a similar domain organization [9]. The N-terminal domains both contain an essential coiled coil motif involved in multimerization while the respective C-terminal domains are required for specific virus binding [21], [22]. Indeed, the C-terminal domain of Fv1 can be replaced with CypA, a molecule that binds HIV-1 CA, resulting in a factor that restricts HIV-1 [23]. TRIM5 has been isolated from a number of mammals including a variety of primates, rabbits and cows [20], [24], [25], [26], [27]. These have been shown to restrict a range of retroviruses from different genera. In particular, TRIM5 from the cotton top tamarin can restrict gammaretroviruses, lentiviruses, spumaviruses and betaretroviruses [24], [28], [29]. Comparison of the target sequences show little identity and although the gammaretroviral, betaretroviral and lentiviral CA molecules show a similar tertiary structure [30] the spumavirus target is folded very differently [31]. Residues in the C-terminal B30.2 domain of TRIM5α that determine viral recognition, and thus restriction specificity, are under strong positive selection [32], [33] and are thought to evolve under pressure imposed by retroviral infection [3], [34], [35]. However in no case have the viruses involved been identified unambiguously [36], [37], [38].

In contrast, changes in Fv1 and the acquisition of its antiviral activity are less well defined. Based on its distribution in different subgenera of *Mus*, it appears that the *Fv1* gene was inserted around 4–7 million years ago [39], [40]. However this finding is somewhat paradoxical because the only known target for Fv1, MLV, probably arose considerably more recently as judged by the distribution of its endogenous forms [41], [42]. What then drove the spread and survival of the *Fv1* open reading frame? Could it be that viruses other than MLV selected for *Fv1*? To address this question we have developed a panel of *Fv1* genes from different mice and investigated their anti-viral activity against a variety of retroviruses. These studies reveal an extraordinary degree of plasticity in the *Fv1* gene as well as two non-MLV viral targets suggesting that a number of different viruses have moulded its evolution.

## Results

### Isolation of *Fv1* from wild mice

To study the evolution of *Fv1*, we set out to clone the gene from a variety of species of *Mus*. Consistent with previous reports [39], [40], it proved possible to clone *Fv1* from multiple species of the subgenus *Mus* as well as single examples of the subgenera *Mus nannomys* and *Mus pyromys* (Table 1). However, we failed to amplify *Fv1* from *Mus coelomys*, *Apodemus* and *Rattus* despite multiple attempts [43], suggesting that the insertion leading to *Fv1* arose about five million years ago, at the time when the ancestors of *Pyromys*, *Nannomys* and *Coelomys* diverged [44]. Sequencing revealed open reading frames in all cases except *Mus mus terricolor (dunni)* and *M. m. cookii* (Figure S1). In *M. m terricolor*, this was due to a single base pair deletion at position 224 that causes a frameshift and premature stop, while in *M. m. cookii*, a base pair transition from C to T at position 650 coupled with a 5 base pair deletion causes the formation of a premature stop codon. Interestingly, in three cases, *M. m. molossinus*, *M. m. spretus* and *M. m. caroli*, 2 different sequences were amplified in reproducible fashion. Pairs clustered together in phylogenetic analyses, suggesting the presence of more than one segregating allele in these subspecies of mice.

**Table 1:** Sources of Fv1 clones.

	Species	Location	DNA source^a^	Abbreviation	Accession number
**PCR Clones**	Lab C57BL/J		TJL	Fv1^b^	X97719
	Lab AKR/J		TJL	Fv1^n^	X97720
	Lab DBA/2J		TJL	Fv1^d^	KF975437
	Lab 129/SvEv		TJL	Fv1^nr^	AY294331
	Lab LG/J		TJL	Fv1^lg^	KF975438
	*M.m.molossinus*	Japan	TJL	MOL	KF975439
	*M.m.bactrianus*	Iran	LGP	BAC	KF975440
	*M.m.castaneous*	India	LGP	CAS1	KF975441
		Thailand	TJL	CAS2	KF975442
	*M.m.spretus*	Spain	TJL	SPR1	KF975443
		France	LGP	SPR2	KF975444
	*M.m.spicilegous*	Yugoslavia	LGP	SPI	KF975445
	*M.m.caroli*	Thailand	TJL	CAR1	KF975446
		Thailand	TJL	CAR2	KF975447
	*M.m.cervicolor*	India?	JMC	CER	KF975448
	*M.m.terricolor*	India	MDTF	DUN	KF975449
	*M.m.cooki*	Thailand	LGP	COO	KF975450
	*M.m.famulus*	India	JMC	FAM	KF975451
	*M.n.minutoides*	Africa	BAM	MIN1	KF975452
	*M.p.platythrix*	India	LGP	PLA	KF975453

a LGP, a gift from Drs Francois Bonhomme and Jean-Louis Guénet, Laboratoire Genome et Populations, Montpellier, France; JMC, a gift from Dr John Coffin, Tufts University, Boston, USA; BAM, a gift from Dr Beverley Mock, NCI, Bethesda, USA; TJL, purchased from the Jackson Laboratory, Bar Harbor, USA; MDTF, *M.dunni* tail fibroblast cells [65].

**Figure 1:**
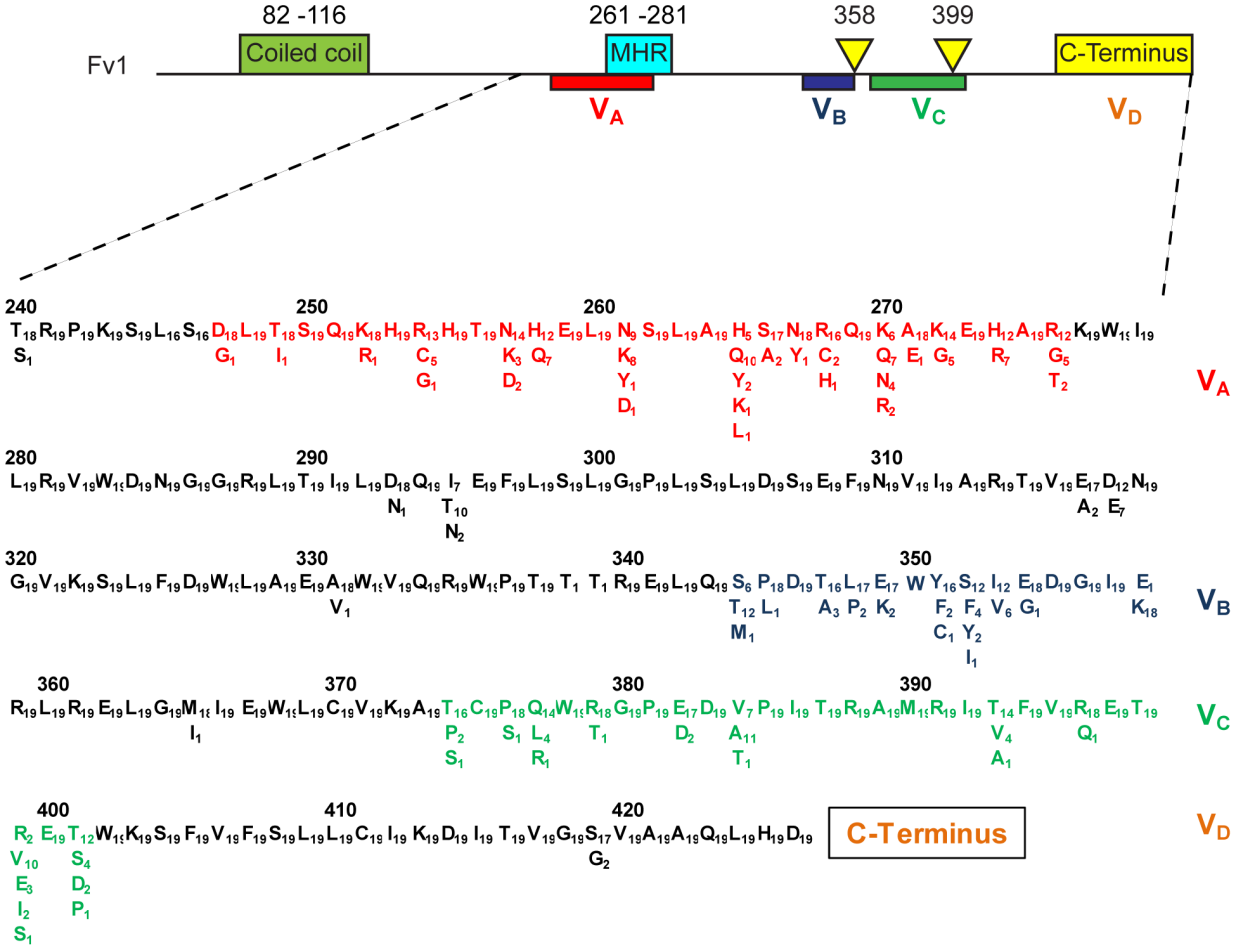
Features of Fv1. At the top of the figure is a schematic of the Fv1 protein showing the relative positions of the previously mapped functional domains including coiled coil and major homology regions as well as the host range specificity regions previously defined by a comparison of Fv1^n^ and Fv1^b^ [48]. Below is shown the positions of four variable regions (V_A–D_), the amino acid differences that define them and the number of times each amino acid occurs. Based on a comparison of 19 mice (Table 1 plus Table 2).

Sequence comparisons reveal that the N-terminal region of *Fv1*, which encodes an extended coiled coil region necessary for restriction activity [23], [45], is well conserved (Figure S1). Compared to *M. m. caroli*, *M. m. famulus*, *Mus nannomys minutoides* and *Mus pyromus platythrix*, all the other Fv1s contained a 3 amino acid insertion near the N-terminus (Figure S1). This change was not important for restriction activity. By contrast, the C-terminal domain shows significant variation in regions important for Fv1 function. Four variable regions, which we designate V_A–D_ can be distinguished (Figure 1). The first variable region (residues 247–276) overlaps a sequence called the Major Homology Region (MHR) that is present in the CA protein of all retroviruses as well as Fv1 [18], [46], [47] and is essential for *Fv1* function [48]. Variable regions B–D (amino acids 345–358, 375–401 and the extreme C-terminus) contain the residues we had previously shown to distinguish the predicted products of the n and b alleles of *Fv1*. These differences are found at amino acids 358, 399 and the very C-terminus of the Fv1 protein where an apparent deletion of 1.3 kb in genomic DNA resulted in a nineteen amino acid length difference [18]; together they appear responsible for the differences in restriction specificity [48]. The present analysis showed that the more divergent mice contained the residues that are found in *Fv1^n^* at positions 358 and 399 but they did not contain the 1.3 kb deletion. This suggested that *Fv1^n^* arose from the progenitor *Fv1*, which was similar in length to *Fv1^b^*, through an internal deletion, while *Fv1^b^* evolved through the substitution of the residues at positions 358 and 399.

**Figure 2:**
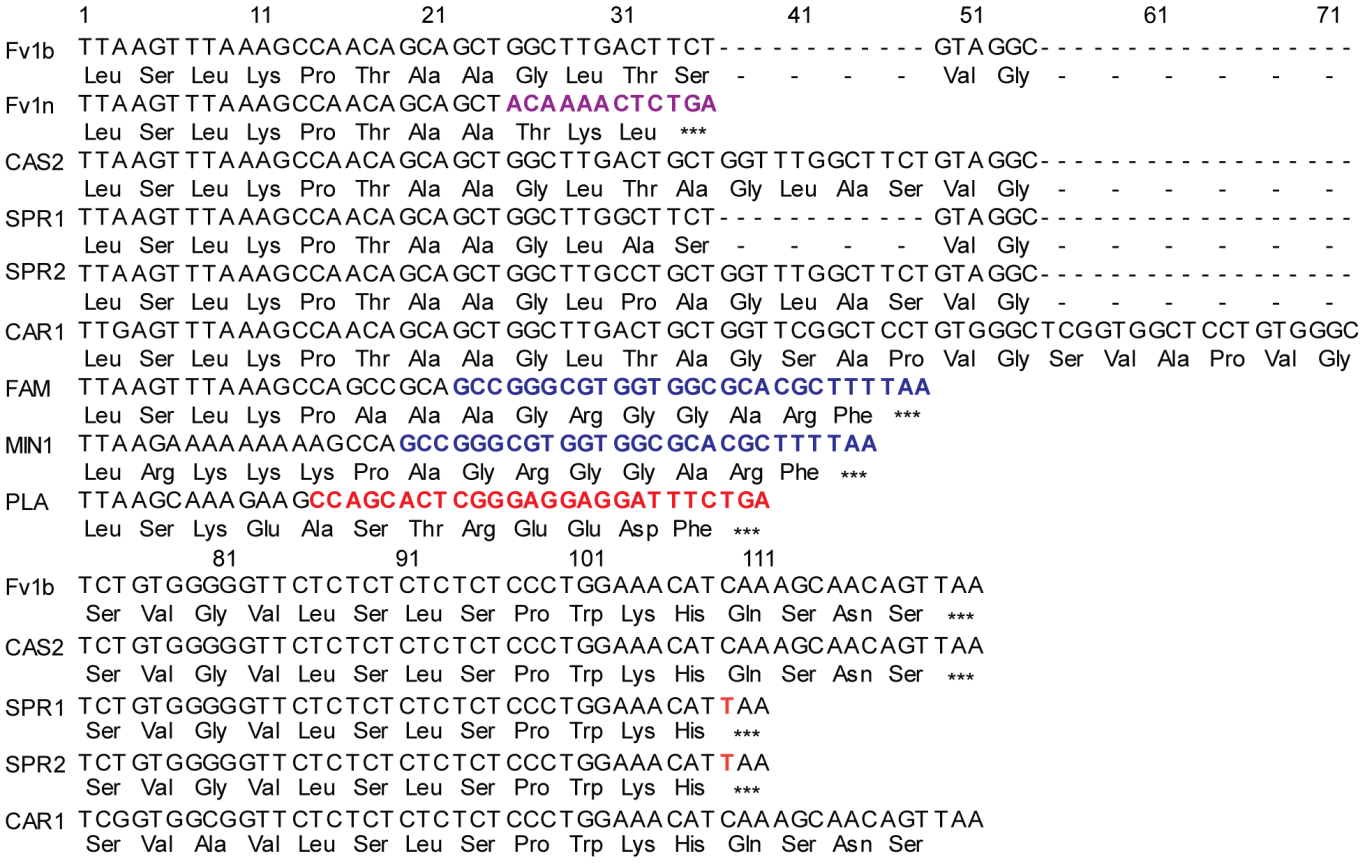
The C-terminal region of selected Fv1 proteins. Nucleic acid and predicted amino acids found at the C-terminus of selected *Fv1* genes. The twelve nucleotides shown in purple for *Fv1^n^* are found 1.3 kb downstream in *Fv1^b^* [18]. Sequences shown in blue (FAM, MIN) correspond to nucleotides 1–27 of the consensus B1 repeat [69]. The sequences in red (PTX) might also come from a rearranged B1 repeat as they correspond to nucleotides 30–44 and 56–65 of the consensus sequence. The nucleotide in orange corresponds to a unique point mutation found in *Mus m. spretus* resulting in a novel stop codon.

Variable regions A–C appear to arise by point mutation but region D shows more significant changes in nucleotide sequence. The three most distantly-diverged mice, *M. n. minutoides*, *M. m. famulus* and *M. p. platythrix* each appear to have B1 repeat sequences inserted, apparently independently, near the deletion site that gave rise to the *Fv1^n^* allele (Figure 2). They contribute the last few amino acids of Fv1 resulting in C-termini that are rather different from either Fv1^n^ or Fv1^b^. Other differences in this region arise from short insertions or deletions perhaps resulting from polymerase slippage during DNA replication. Thus the clones, SPR1 and SPR2, we amplified from *M. m. spretus* of French and Spanish origins differed by four amino acids; the same difference also seen between Fv1^b^ and CAS2 (Figure 2).

### Different restriction activities of wild mice Fv1

**Figure 3:**
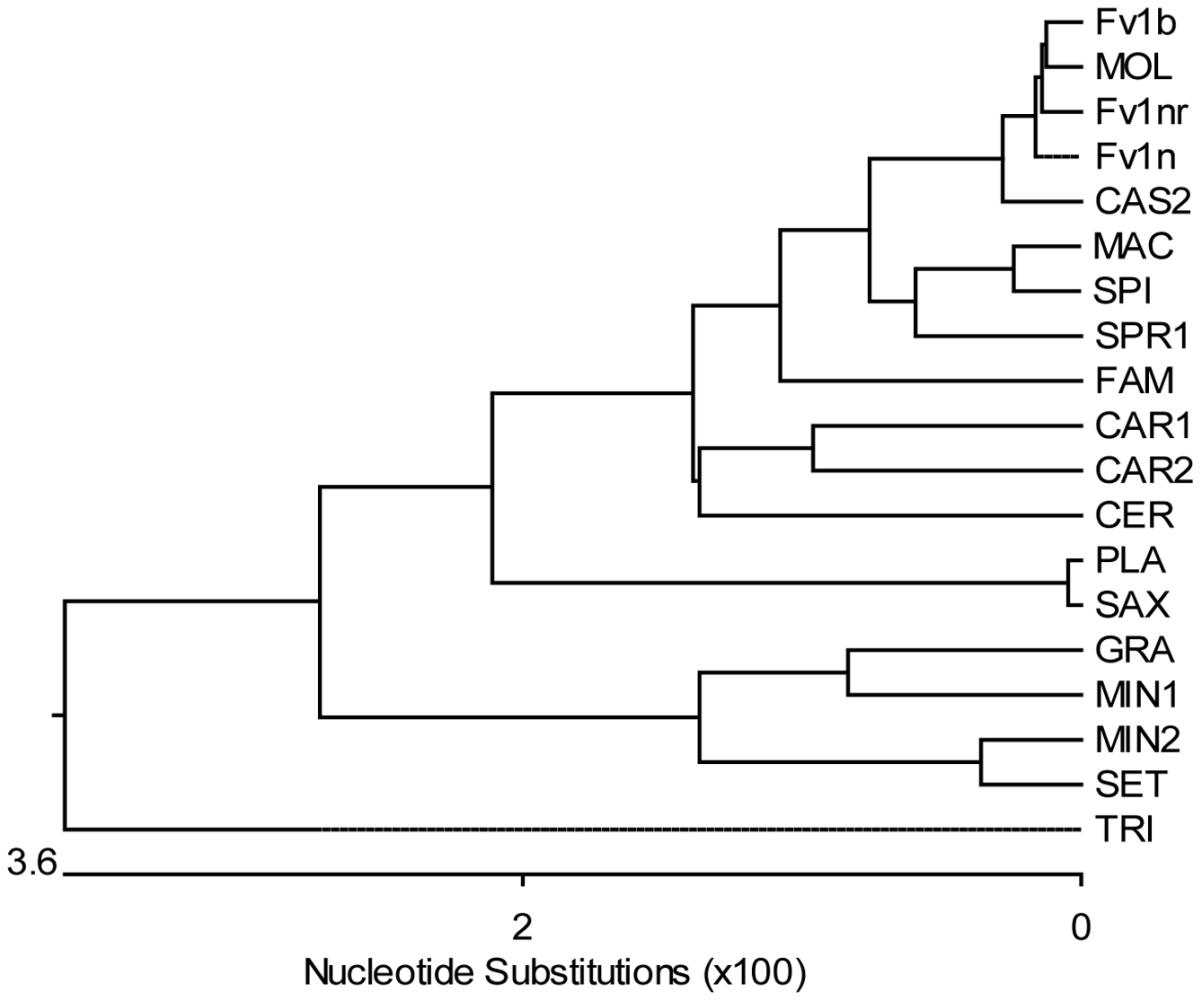
Phylogenetic tree of *Fv1* sequences. The tree was generated from the open reading frames listed in Tables 1 and 2 (bases 1 to 1278 in *Fv1^b^*) using the MegAlign programme from the DNASTAR Lasergene package. The highly divergent C-terminus was excluded from the analysis and the aspartic acid residue at position 426 (Fv1^b^ numbering), which was the last residue conserved in all the sequences, was chosen as the cut-off point. The number of substitution events is shown at the bottom of the tree while the distances between sequence pairs is represented by the length of the branch pairs. The distance values were calculated using the Kimura distance formula that takes into account the number of non-gap mismatches and silent substitutions.

By analogy with other restriction factors it seems likely that the variation seen in *Fv1* arose by selection and this would be reflected in changes in restriction specificity. To test this we examined the restriction properties of *Fv1* we had cloned from different mice (Table 1) as well as a number of genes synthesized on the basis of published sequences (Table 2) [40], [49]. A tree based on these sequences is shown in Figure 3; it shows good agreement with the accepted phylogeny of genus *Mus* [50]. The *Fv1* genes were introduced into pLgatewayIYFP and tested for restriction of different MLVs using a two-colour FACS assay [19], [51]. The results are shown in Table 3.

**Table 2:** Sources of Fv1 synthesized clones.

Species	Location	DNA source^a^	Abbreviation	Accession number
*M.m.macedonicus*	Bulgaria?	Yan	MAC	FJ603564
*M.n.minutoides*	Africa	Yan	MIN2	FJ603554
*M.n.gratus*	Uganda	Yan	GRA	FJ603556
*M.n.setulosis*	Kenya	Yan	SET	FJ603555
*M.n.triton*	Uganda	Yan	TRI	FJ603557
*M.p.saxicolor*	India	Yan	SAX	FJ603560

a Yan, synthesized based on sequence published in [40].

**Table 3:** Restriction activity of various Fv1s against different MLVs.

	Virus					
Fv1 allele	N-MLV	B-MLV	NB-MLV	N-MLV D82N	N-MLV H114R	N-MLV L117H
Fv1^b^	**0.09±0.01**	*0.48±0.02*	**0.16±0.01**	**0.09±0.01**	**0.16±0.01**	**0.12±0.02**
Fv1^n^	1.18±0.07	**0.11±0.01**	1.14±0.01	1.20±0.04	1.15±0.01	1.15±0.04
MOL	**0.20±0.03**	**0.22±0.02**	1.07±0.01	**0.21±0.01**	1.24±0.01	1.19±0.01
CAS2	1.17±0.03	1.15±0.01	1.13±0.03	1.24±0.07	1.16±0.03	1.19±0.01
SPI	1.18±0.04	1.18±0.06	1.16±0.05	1.20±0.01	1.10±0.02	1.16±0.06
SPR1	**0.12±0.01**	**0.13±0.01**	1.17±0.01	0.89±0.01	1.07±0.02	*0.68±0.07*
CAR1	1.21±0.07	1.15±0.03	1.17±0.01	1.31±0.02	1.24±0.02	1.31±0.06
CAR2	1.02±0.01	1.13±0.01	1.16±0.03	1.21±0.01	1.14±0.01	1.20±0.03
CER	0.90±0.01	1.15±0.01	1.15±0.01	1.17±0.02	1.20±0.08	1.16±0.06
FAM	*0.31±0.01*	*0.36±0.02*	*0.38±0.03*	*0.37±0.01*	0.73±0.04	*0.29±0.01*
MIN1	**0.23±0.06**	**0.18±0.02**	**0.24±0.01**	**0.17±0.02**	**0.21±0.01**	**0.22±0.02**
PLA	1.09±0.05	1.08±0.01	1.17±0.04	1.11±0.01	0.81±0.01	1.19±0.04
MAC	**0.18±0.01**	1.24±0.02	1.24±0.01	n.d.	n.d.	n.d.
MIN2	**0.04±0.01**	**0.04±0.01**	**0.10±0.01**	n.d.	n.d.	n.d.
GRA	1.22±0.03	1.20±0.01	1.25±0.01	n.d.	n.d.	n.d.
SET	**0.10±0.01**	**0.06±0.01**	1.25±0.02	n.d.	n.d.	n.d.
TRI	1.12±0.01	*0.45±0.01*	1.31±0.01	n.d.	n.d.	n.d.
SAX	1.32±0.02	1.30±0.02	1.30±0.03	n.d.	n.d.	n.d.

As previously shown, the *Fv1^n^* gene restricted B-MLV but not N- or NB-MLV while *Fv1^b^*, when expressed at protein levels seen in transduced cells, restricted N- and NB- MLV and also had a weak activity against B-MLV [51]. The *Fv1* gene from *M. m. molossinus* and *M. m. spretus* restricted both N- and B-MLV but not NB-MLV, suggesting that they could be similar to *Fv1^nr^* [16]. Hence, we also examined three N-MLV CA variants (D82N, H114R and L117H) that confer resistance to *Fv1^nr^* [16]. Variants N-MLV H114R and N L117H, which we have found to be NR tropic, were also resistant to both the Fv1MOL1 and Fv1SPR1 proteins. In contrast, N-MLV N82D was restricted by Fv1MOL1 but not by Fv1SPR1, suggesting that Fv1MOL1 is subtly different from Fv1^nr^. We have cloned the *Fv1^nr^* gene from 4 different strains of mice (129SvEv, NZB/B1NJ, NZW/LacJ and RF/J); all contained a single nucleotide change compared to *Fv1^n^*, causing a serine to phenylalanine substitution at residue 352. While *Fv1MOL1* also encoded phenylalanine at position 352, *Fv1SPR1* possesses a serine at the corresponding position. Taken together, these results suggest that other changes could also be involved in determining the nr-specificity. Perhaps surprisingly, *Fv1* from two closely related species, *M. m. castaneus* and *M. m. spicelegus* lacked perceptible Fv1 activity against MLV.

Other *Fv1* genes displayed a variety of restriction phenotypes. Fv1 from two asian members of the *Mus* subgenus, *M. m. caroli* and *M. m. cervicolor*, lacked Fv1 activity directed against MLV as did the two members of the *Pyromys* subgenus that we tested, *M. p. platythrix* and *M. p. saxicolor* (Table 3). By contrast two members of the *Nannomys* subgenus, *M. n. ninutoides* and *M. n setulosis* were active with the *M. n. minutoides* clones restricting all six MLVs tested. The *M. m. famulus* sample, whose position in *Mus* phylogenetic trees is relatively poorly defined, showed weak activity against N, B, and NB-tropic MLVs. Thus more than half of the *Fv1* genes with intact open reading frames did not seem to have any activity against MLV, the target that defines the *Fv1* gene, even though they were expressed at similar levels to restricting genes in transduced cells (Figure S2). Further, the extent and specificity of restriction of different MLVs varies significantly. Clearly, the properties of the restriction gene have changed since the gene first became part of the mouse germ line but whether MLV alone was responsible for selecting such changes remained an open question.

### Novel restriction specificities of wild mice Fv1

Prompted by the example of TRIM5α that can restrict multiple genera of retrovirus [24], [29], we decided to investigate the hypothesis that non-MLV retroviruses might play a role in shaping the evolution of *Fv1*, by testing a number of different retroviral vectors for restriction by *Fv1* from wild mice. These included other gammaretroviruses like Gibbon Ape Leukemia Virus (GALV), Feline leukemia Virus (FeLV) and Porcine Endogenous Retrovirus-A (PERV-A), lentiviruses such as HIV-1, HIV-2, SIVmac, Equine Infectious Anemia Virus (EIAV) and Feline immunodeficiency Virus (FIV), as well as foamy viruses including Prototypic Foamy Virus (PFV), Simian Foamy Virus (SFV) and Feline Foamy Virus (FFV). Some of these results are presented in Table 4. The data show that *Fv1* from *M. m. caroli*, that lacked activity against MLV, restricted FFV strongly and PFV weakly. Moreover, *Fv1* from *M. m. spretus*, which restricted N- and B-MLV, and from *M. m. macedonicus*, which inhibited N-MLV, were also active against the lentivirus EIAV. By contrast GALV, FeLV, PERV-A, SFV, HIV-1, SIVmac and FIV were not restricted by any of the *Fv1* genes in the panel (Table 4 and data not shown). Formally it remains possible that the novel specificities observed result from over expression. Unfortunately, no cell lines expressing Fv1CAR1 and Fv1SPR1 at endogenous levels are available, precluding a direct test of this idea. However we are not aware of any examples of complete restriction of novel viruses resulting from such a mechanism.

**Table 4:** Restriction activity of various Fv1s against different viruses.

	Virus				
Fv1 allele	PFV	SFV	FFV	EIAV	HIV-1
Fv1^b^	0.99±0.02	1.10±0.02	1.00±0.02	*0.52±0.01*	1.12±0.01
Fv1^n^	1.08±0.06	1.02±0.01	1.06±0.01	1.17±0.04	1.12±0.01
MOL	1.11±0.07	1.08±0.02	1.04±0.01	1.18±0.12	1.16±0.05
CAS2	1.10±0.07	1.05±0.01	1.06±0.02	1.28±0.06	1.14±0.01
SPI	1.00±0.03	1.05±0.02	1.03±0.02	*0.70±0.06*	1.13±0.01
SPR1	1.04±0.03	1.05±0.01	1.02±0.01	**0.20±0.01**	*0.70±0.01*
CAR1	*0.33±0.01*	0.92±0.02	**0.13±0.01**	1.17±0.09	1.31±0.01
CAR2	1.36±0.04	1.09±0.01	1.10±0.02	1.28±0.12	1.19±0.04
CER	1.04±0.02	1.13±0.03	1.09±0.02	0.81±0.02	1.14±0.01
FAM	1.05±0.01	1.08±0.01	1.06±0.01	0.96±0.03	1.16±0.01
MIN1	1.02±0.01	1.10±0.01	1.31±0.02	1.44±0.10	1.21±0.03
PLA	1.00±0.03	1.03±0.02	1.04±0.01	1.08±0.01	1.10±0.01
MAC	1.05±0.03	0.99±0.01	1.01±0.03	**0.21±0.01**	0.96±0.01
MIN2	1.00±0.02	1.04±0.01	1.00±0.02	1.08±0.04	1.10±0.02
GRA	0.91±0.01	1.03±0.01	0.96±0.01	0.97±0.01	1.00±0.01
SET	0.81±0.03	1.01±0.01	0.99±0.01	0.99±0.04	1.09±0.02
TRI	0.88±0.05	1.04±0.02	0.98±0.02	0.96±0.01	1.17±0.04
SAX	1.01±0.02	1.02±0.01	1.05±0.09	0.95±0.03	1.10±0.01

**Figure 4:**
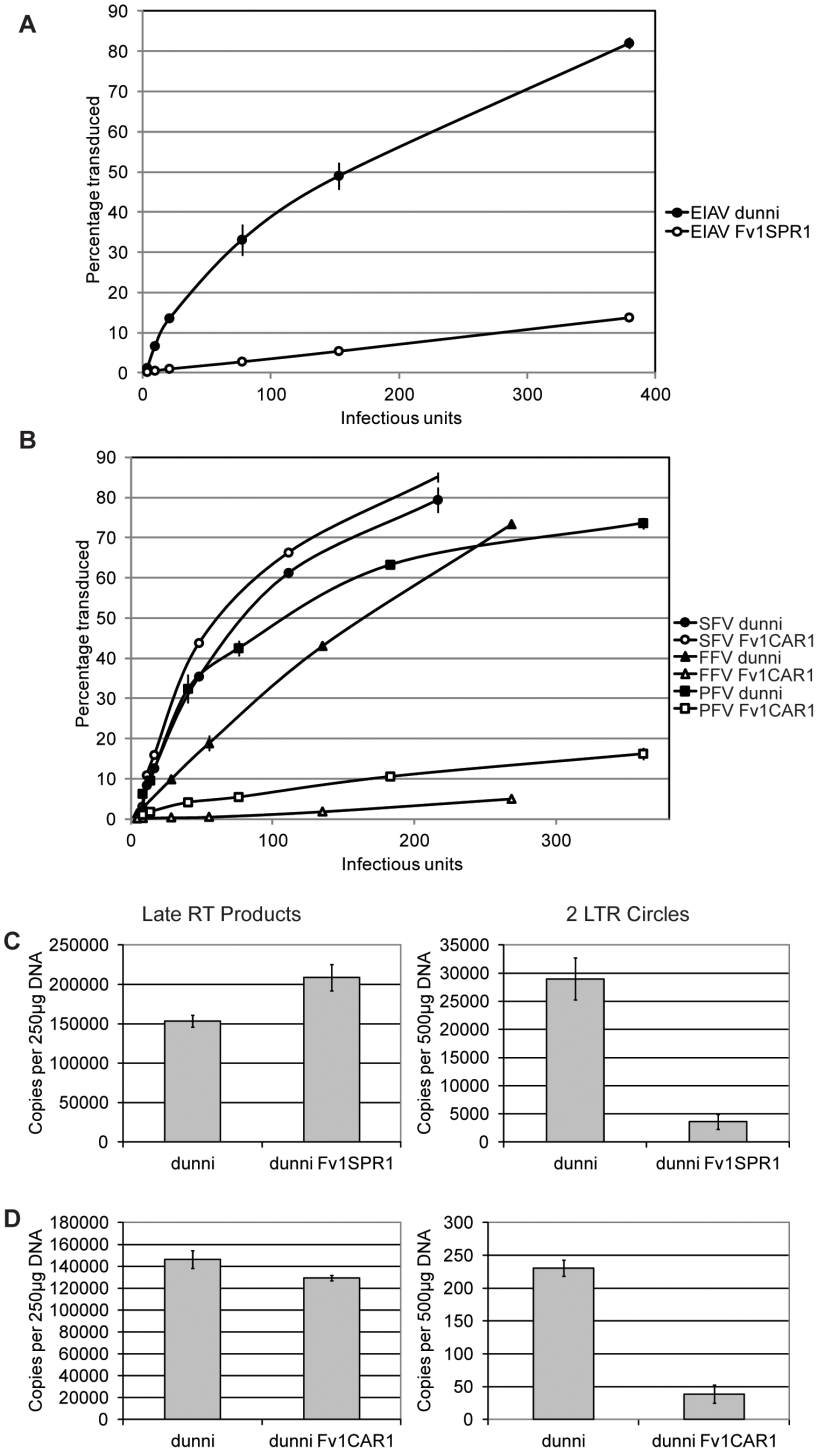
Staging restriction blocks in novel *Fv1*s. MDTF cells were transduced with *Fv1CAR1* and *Fv1SPR1*, then stable Fv1-expressing cell lines selected and tested for restriction of virus replication by FACS (A, B) or by PCR to measure viral DNA synthesis or formation of circular viral DNA containing two LTRs (C, D).

To further characterize restriction mediated by *Fv1CAR1* and *Fv1SPR1* stable cell lines were derived by transducing MDTF cells with retroviral vectors carrying these genes and selecting for G418-resistant single cell clones. These cell lines were used in virus titrations by measuring the percentage of transduced cells by FACS with different amounts of virus (Figure 4A,B). As expected, the titre of EIAV was dramatically reduced in the cell line expressing Fv1SPR1 compared to the untransduced MDTF control (Figure 4A). Similarly titres of FFV and PFV were greatly reduced in MDTF cells expressing *Fv1CAR1* compared to untransduced while titres of SFV were unaffected by the presence of the *Fv1* gene (Figure 4B). These results confirm the observations made with the 2 colour FACS assay that *Fv1* from some wild mice can restrict non-MLV retroviruses.

*Fv1* is thought to interfere with MLV replication by preventing nuclear import of newly synthesized viral DNA [9]. To test whether this was also true for EIAV and FFV, we examined the fate of viral DNA in restricting cell lines. Testing EIAV replication in *Fv1SPR1* cells shows no inhibition of reverse transcription as measured by levels of newly synthesized late DNA products (Figure 4C). However levels of 2-LTR circles, which are thought to form only after nuclear entry [52], are substantially reduced suggesting a block in nuclear uptake. In *Fv1CAR1* cells a reduction in FFV 2-LTR circles, with no change in late RT products, was also observed (Figure 4D), again consistent with a block in nuclear import. However, interpretation of these data is complicated by the fact that the majority of FFV DNA synthesis is thought to occur in the producer cells [53]. Nevertheless, it appears likely that Fv1 is acting to block lentivirus and foamy virus replication at the same stage in the viral life cycle as seen with MLV.

### Mapping the specificity determinants of the novel restriction activities

**Figure 5:**
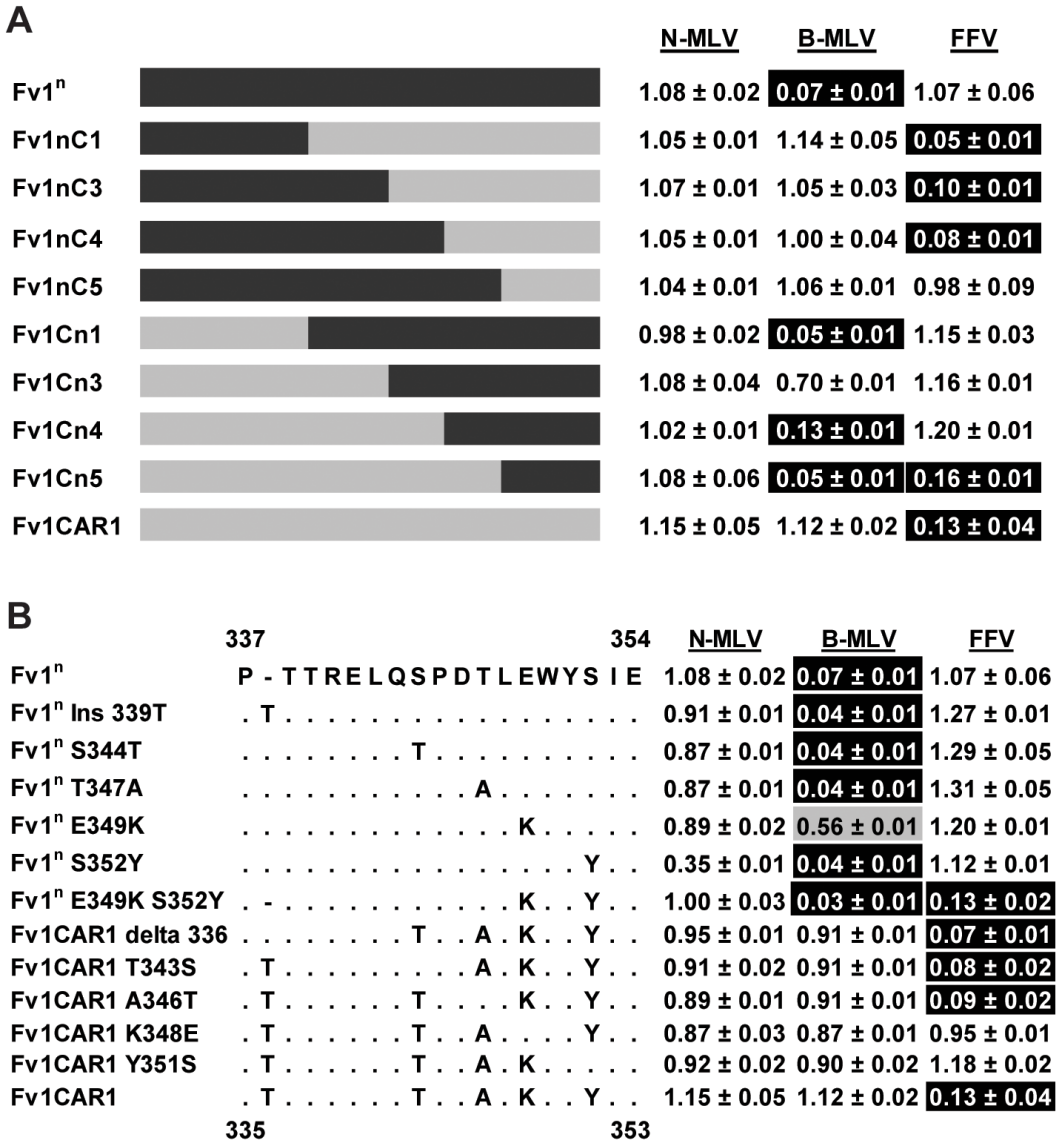
Mapping the determinants of FFV restriction by Fv1 from *M. m. caroli*. (A) Analysis of restriction by chimeric Fv1 constructs. (B) Analysis of restriction by site directed mutant forms of Fv1.

To identify the specificity determinants of these novel restriction activities, chimeric *Fv1* genes were constructed and tested for restriction. To look at FFV restriction, we made chimeras between *Fv1CAR1*, which restricted only FFV, and *Fv1^n^*, which restricted B-MLV. Schematic views of the constructs made and the corresponding restriction data are shown in Figure 5A. Replacement of a C-terminal fragment of Fv1^n^ (from residue 318) with the corresponding fragment from Fv1CAR1 generated a chimera (Fv1nC4) capable of restricting FFV. Replacement with a shorter fragment starting from residue 353 (Fv1nC5) was insufficient to confer restriction, suggesting that the determinants of FFV restriction were found between residues 316 and 352 of Fv1 from *M. m. caroli*. In the reciprocal chimeras, replacing the small C-terminal segment of Fv1CAR1 beginning from residue 352 with that from Fv1^n^ did not result in any loss of activity against FFV. However, when a larger fragment beginning at residue 316 was replaced, activity was lost, confirming the presence of the determinants of FFV restriction within the region of Fv1CAR1 between residues 316 and 352. Within this region, there are 5 residues that differ between Fv1^n^ and Fv1CAR1. These were systematically changed to identify the residues involved in specificity determination (Figure 5B). No single change could endow Fv1^n^ with the ability to restrict FFV (Figure 5B). However two single changes at positions 349 and 352 of Fv1CAR1 resulted in loss of FFV restriction. We therefore mutated both these positions in Fv1^n^ to the corresponding amino acids found in Fv1CAR1. This generated a construct (Fv1^n^E349KS352Y) capable of restricting FFV. Taken together, these results indicate that both lysine 349 and tyrosine 352 in Fv1 from *M. m. caroli* are crucial for FFV restriction. We had previously shown that MLV recognition maps downstream of this region [48]; it was therefore interesting to see that Fv1^n^E349KS352Y (and chimera Fv1Cn5) could recognize both B-MLV and FFV in an additive fashion.

**Figure 6:**
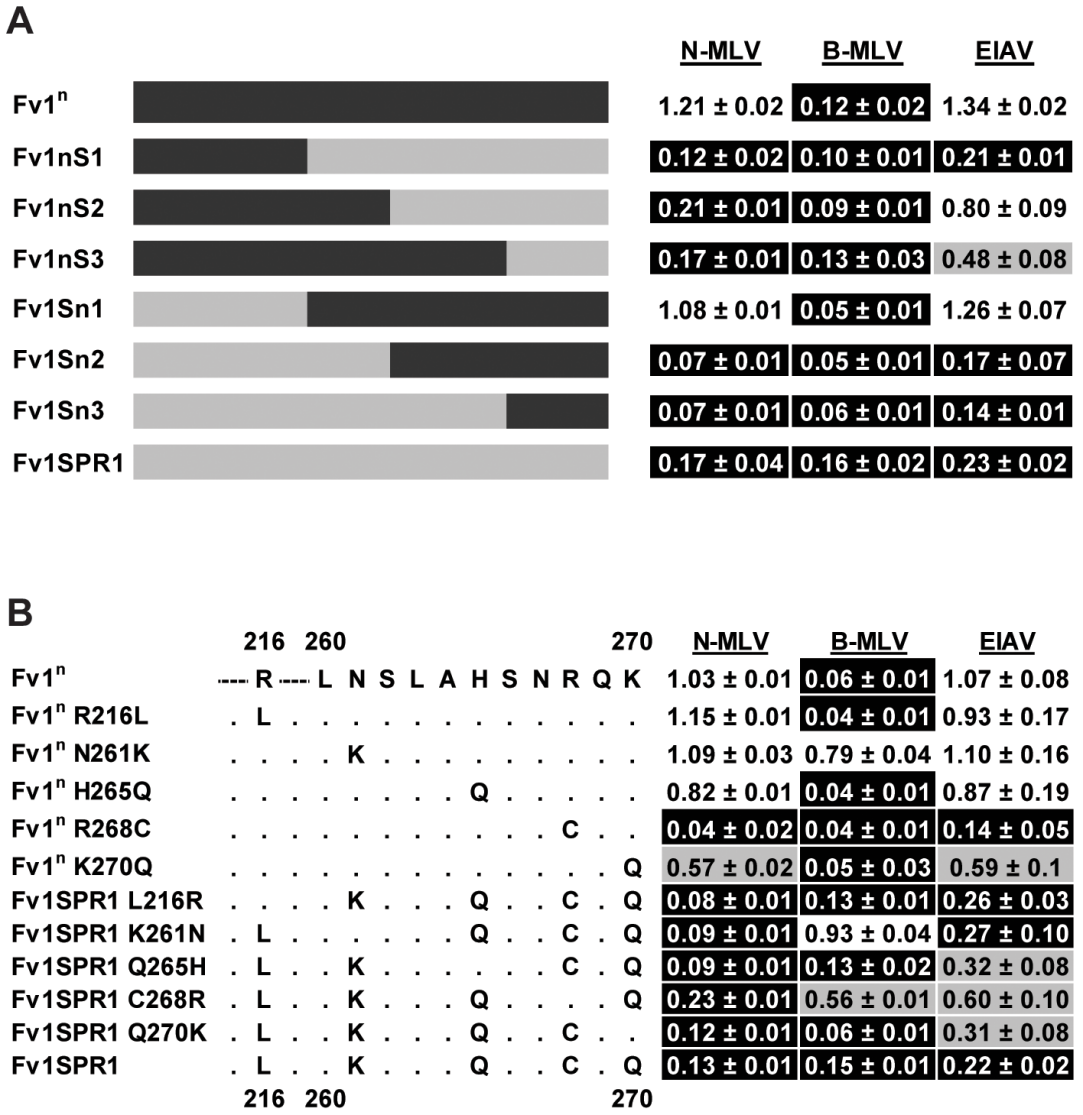
Mapping the determinants of EIAV restriction by Fv1 from *M. m. spretus*. (A) Analysis of restriction by chimeric Fv1 constructs. (B) Analysis of restriction by site directed mutant forms of Fv1.

To examine EIAV restriction by Fv1 from *M. m. spretus*, a second set of chimeras was made between *Fv1SPR1*, which restricts N-MLV, B-MLV and EIAV, and *Fv1^n^*, which only restricts B-MLV. Restriction of EIAV was seen with chimeras only when amino acids from positions 191 and 271 were derived from Fv1SPR1 (Fig. 6A) suggesting that the determinants of EIAV restriction lay between these residues. Interestingly, the determinants for MLV restriction were slightly different from those of EIAV. Replacing a short segment of C-terminus of Fv1^n^ (from residue 366) with that from Fv1SPR1 in Fv1nS3 was sufficient to confer restriction of N-MLV, suggesting that this region contained determinants of N-MLV restriction. However, a reciprocal change in Fv1SPR1 (Fv1Sn3) did not abolish N-MLV restriction. It was only when a C-terminal segment beginning with residue 191 was replaced from Fv1SPR1 (Fv1Sn1) that the restriction of N-MLV was lost. This suggested that additional requirements for N-MLV restriction were found between residues 191 and 271 of Fv1 from *M. m. spretus*, perhaps overlapping with those that determined EIAV restriction.

There are 5 differences between Fv1^n^ and Fv1SPR1 in the segment between residues 191 and 271 (Figure 6B). To identify the residues involved in restriction, site-directed mutagenesis was employed to change the residues in Fv1^n^ to those present in Fv1SPR1. Reciprocal mutations were also made in Fv1SPR1. These mutants were tested for restriction of EIAV, N- and B-MLV. The substitution from arginine to cysteine at position 268 in Fv1^n^ was sufficient to confer the ability to restrict both N-MLV and EIAV. The reciprocal change in Fv1SPR1 resulted in a partial reduction in restriction of all three viruses. These results indicated that residue 268 was the major determinant of EIAV restriction by Fv1SPR1 and had an influence on MLV restriction but that other neighbouring residues were also important. A lysine to glutamine change at residue 270 in Fv1^n^ resulted in low but reproducible restriction of EIAV and N-MLV though the reciprocal change in Fv1SPR1 had little effect. Interestingly, substitution of the residue at position 261 in both Fv1^n^ and Fv1SPR1 seemed to abolish the restriction of B-MLV, indicating that this residue was involved in the interaction with B-MLV. We conclude that residues 261, 268 and 270 in Fv1 from *M. m. spretus* are all involved in virus recognition. However, it would appear that recognition of EIAV by Fv1 from *M. m. macedonicus* has arisen in a different manner as it contains arginine rather than cysteine at position 268.

## Discussion

In this study of *Fv1* evolution we have demonstrated that Fv1 shows substantial sequence variation in its C-terminal half, the region of the protein thought to contain determinants of restriction specificity. In addition we have shown that Fv1 is capable of restricting viruses other than its previously defined targets and identified the sequence variation responsible for these novel targets. We note that some *Fv1* alleles do not appear to possess an associated restriction activity; it would be of considerable interest to determine whether they recognize other targets.

**Figure 7:**
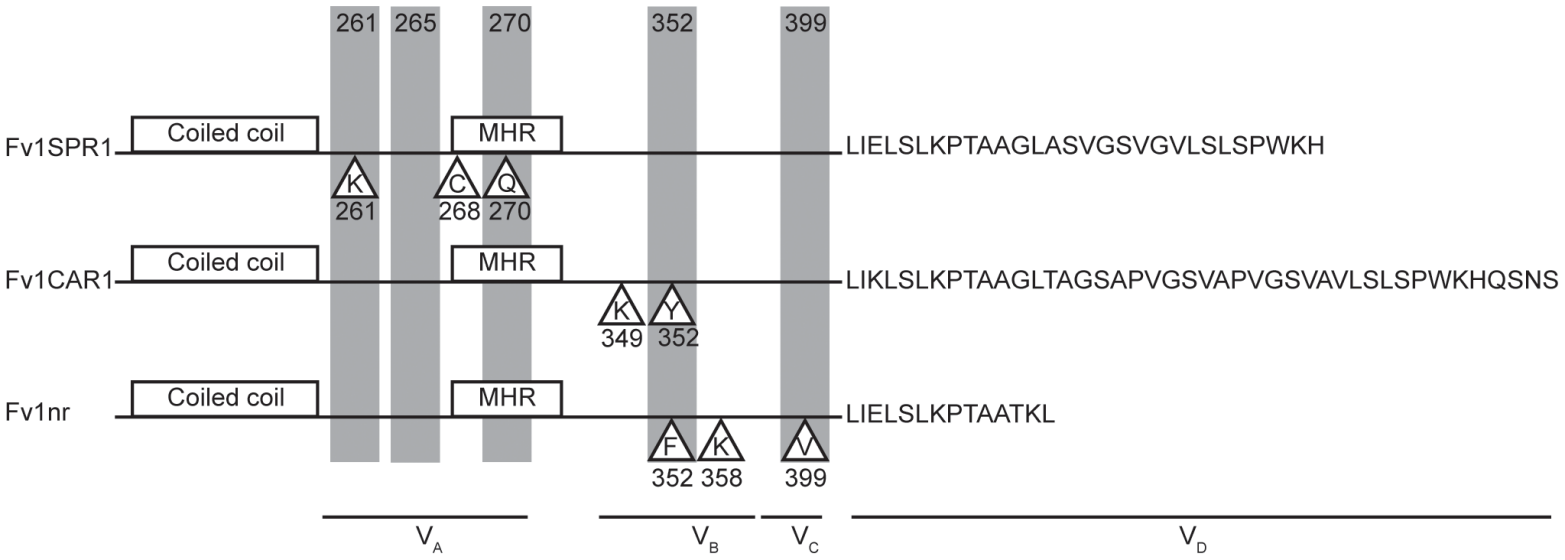
Properties of the variable regions of Fv1. Proteins corresponding to the Fv1SPR1, Fv1CAR1 and Fv1^nr^ alleles are illustrated. Shaded positions indicate amino acids showing positive selection [40], residues in triangles have been implicated in restriction specificity (this paper, [16], [48]).

A previous study had identified six codons, specifying Fv1 amino acids 261, 265, 270, 362, 299 and 401, that show evidence for positive selection during the course of *Mus* evolution [40]. These represent potential sites of interaction between Fv1 and its target viruses. Combining these data with our previous studies of Fv1 specificity [16], [48], it seems reasonable to conclude that the four variable regions defined in Figure 1 constitute four domains collectively or individually involved in target selection and binding (Figure 7). Thus VRA (amino acids 247–276) includes the positively selected residues 261, 265 and 270 as well as three residues, 261, 268 and 270, shown to be important for EIAV restriction by Fv1SPR1 while VRB (amino acids 345–358) has positively selected amino acid 352, amino acids 349 and 352 important for FFV recognition by Fv1CAR1 as well as residues 352 and 358 important for NR- and N- versus B-tropism, respectively [16], [48]. Variable region C (amino acids 375–401) contains positively selected amino acids 399 and 401 while residue 399 was also implicated in determining N- versus B-tropism [48]. The nature of the length variation at the C-terminus precludes computational analysis for positive selection; nevertheless functional studies [48] provide compelling evidence that this region can also alter restriction specificity.

We have previously noted that CA binding restriction factors Fv1 and TRIM5α share certain design features despite lack of sequence similarity [9]; the present study strengthens this analogy. Both factors possess an N-terminal coiled-coil region allowing dimer formation. They also contain other sequences facilitating the formation of higher order multimers. Both contain a C-terminal domain responsible for virus binding that can be substituted with the cellular CA binding cyclophilin A protein to give a fusion protein capable of restricting HIV-1 and other lentiviruses [23], [54]. We now provide evidence that the CA binding domain of Fv1, like TRIM5α [24], [32], [55], [56], appears to comprise multiple variable regions, showing attributes of positive selection, implying virus driven evolution [3]. Further, Fv1 is capable of recognizing multiple genera of retrovirus. It seems possible that the ability to recognize multiple viruses by low affinity binding with avid binding provided by multimerisation [57] represents a common theme in restriction factor design. Further insights into the interaction between virus and restriction factor requires detailed structural information; unfortunately both Fv1 and TRIM5α are relatively recalcitrant to such studies.

The origin of *Fv1* remains unclear. It is only present in *Mus* and appears related to the *gag* gene of the endogenous retrovirus family ERV-L [18], [47]. This suggests that *Fv1* might be derived from an endogenous retrovirus following the loss of both LTRs and *pol* coding sequences [58]. Interestingly a significant increase in MERV-L copy number took place at around the time of the separation of *Mus* subgenera [59], the time when *Fv1* became part of the *Mus* germline. However sequence alignments indicate that Fv1 and MERV-L share only 43% amino acid identity whereas the different genomic MERV-L elements are much more closely related to one another (<5% nucleic acid divergence). BLAST searches of the NCBI non-redundant genome databases reveal no sequences intermediate between *Fv1* and *MERV-L*. This suggests that *Fv1* might be derived from an exogenous virus related to ERV-L that has not made its home as an intact ERV, at least not in any species so far sequenced, and may no longer exist in infectious form. As such *Fv1* might be the last remnant of an ancient extinct virus, or paleovirus [2]. Unfortunately this inability to identify the proximal precursor for Fv1 prevents us from determining whether or not the original transgene showed restriction activity and, if so, against which virus.

**Figure 8:**
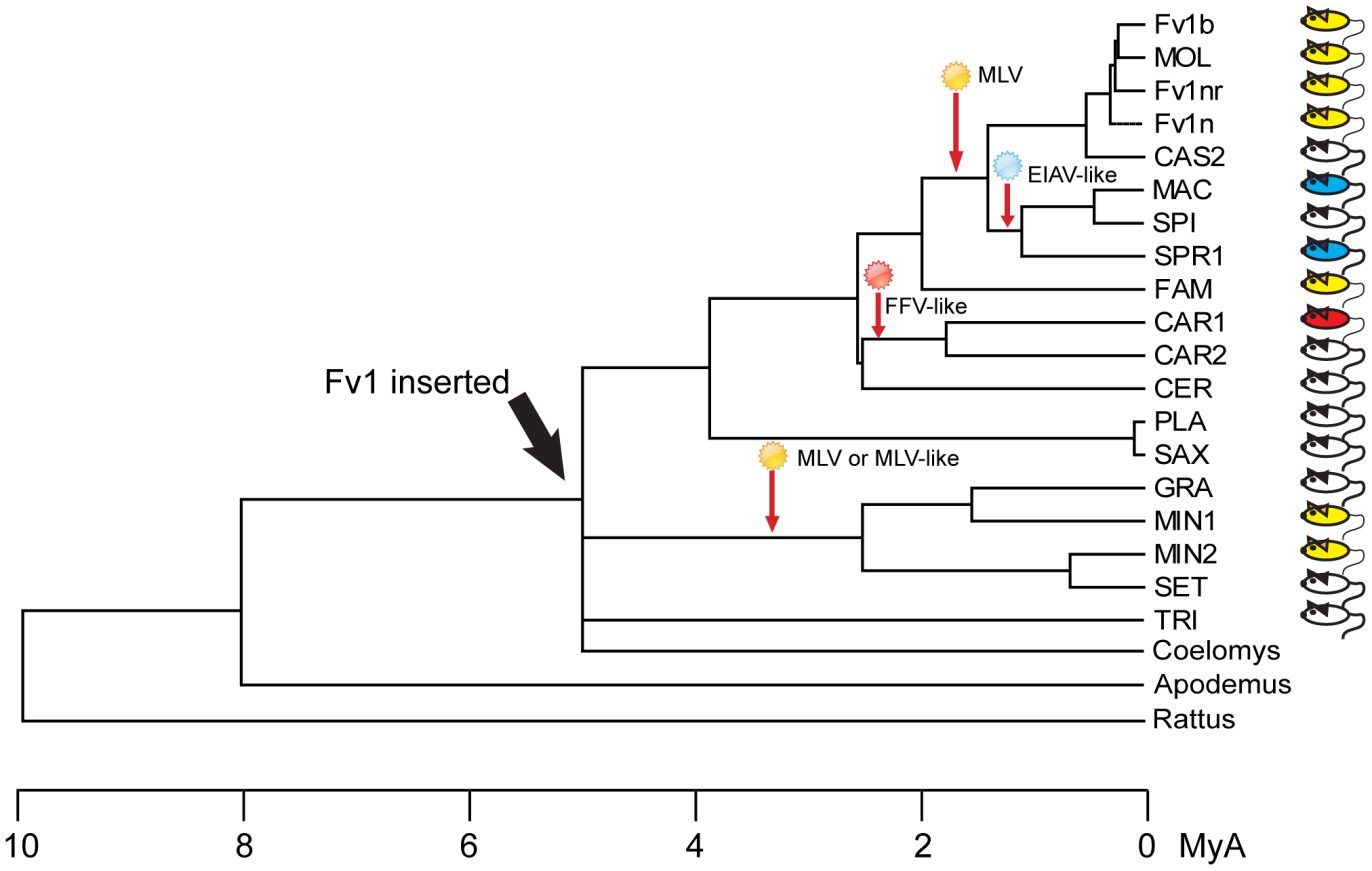
Events in the evolution of the *Fv1* gene. A phylogenetic tree showing the approximate times of *Fv1* acquisition and hypothetical virus infections leading to selection of new retroviral restriction activities. Colored mice indicate *Fv1* activity against at least one virus.

The selection and continuing existence of the Fv1 open reading frame implies that it provides an evolutionary advantage, presumably by providing protection against retroviral infection. The observation of multiple restriction specificities suggests that a variety of unknown viruses have contributed to this process. Taken together with frequent genetic changes to inactivate [60] or block MLV receptors [61], these data imply that multiple virus epidemics have occurred in the course of mouse evolution [62]. One might postulate that at least four significant virus exposures have occurred during *Mus* evolution (Figure 8). One took place after the divergence of *Nannomys*; a second occurred in *M. m. caroli*; a third in mice in countries surrounding the Mediterranean Sea and a fourth in the *Mus musculus* subfamily. In turn this prompts the question of how the current properties of a restriction factor reflect the properties of the viruses involved in selection, a question that is as relevant for TRIM5α as for *Fv1*. Specifically one might ask whether the ability to restrict one genus of retrovirus reflects prior exposure to that kind of virus. An affirmative answer might resolve the vexed question of whether foamy viruses have deleterious effects on their hosts [63], possibly as co-pathogens [64] since both *Fv1* (this paper) and TRIM5α [29] have evolved to see one or more such virus. Alternatively, changes selected by, say, a gammaretrovirus like MLV, might fortuitously result in recognition of a lentivirus like EIAV or a foamy virus like FFV. In light of the shorter generation time of mice compared to primates *Fv1* could provide a more useful system for studying evolution of restriction specificity than does TRIM5α. The observation of multiple alleles of *Fv1* might also suggest that selection is an ongoing process offering opportunities for experimental analysis. In particular, the evolution of restriction activity against the lentivirus EIAV, which appears to have happened in two different ways in *M. m. spretus* and *M. m. macedonicus* as well as the kind and source(s) of the virus(es) involved would appear worthy of more detailed investigation.

## Materials and Methods

### Fv1 cloning

Genomic DNA samples for *Mus musculus* laboratory mouse strains C57BL/6J, AKR/J, DBA/2J, 129/SvEv, and LG/J, *M. m. spretus* (M. spretus), *M. m. caroli* (Mus caroli), *M. m. molissinus* (MOLD/Rk) and *M. m. castaneous* were purchased from the Jackson Laboratory. Genomic DNA from *M. p. platythrix*, *M. m. cookii*, *M. m. spicilegus*, *M. m. spretus*, *M. m. castaneous* and *M. m. bactrianus* were gifts from Dr. F. Bonhomme (Laboratoire Genome et Populations, Universite de Montpellier II, CNRS). *M. n. minutoides* genomic DNA was a gift from Dr. B. Mock (National Cancer Institute, NIH), while *M. m. famulus* and *M. m. cervicolor* genomic DNA were gifts from Dr. John Coffin (Tufts University School of Medicine, Boston). *M. m. terricolor* (dunni) genomic DNA was prepared from a *Mus dunni* tail fibroblast (MDTF) line [65] using the DNeasy blood and tissue kit (Qiagen). The *Fv1* ORF was PCR amplified from mouse genomic DNA using primers PL80 and GT17 (see Table S1 for primer sequences) that permit the amplification of a sequence starting from 3056 bp upstream of the start codon of *Fv1* to 2684 bp downstream of the start codon. Sequence analysis of this region from in-bred mice identified 2 SacI sites downstream of the PL80 primer-binding site, while GT17 contained a SalI site. The PCR products were hence cloned initially as SacI/SalI fragments into M13 phage and sequenced. Subsequent subcloning of Fv1 ORFs was carried out following amplification with the primers GatewayFv1F and Gateway Fv1rev. The PCR product was used in a second amplification reaction with primers UniversalF and UniversalRev to attach the attB sites to the ends of the fragment. This was then inserted into pDNR221, which is an entry vector to the Gateway Cloning system, using BP clonase (Invitrogen). Finally, the entry clone was used in a LR reaction with LR clonase to insert the *Fv1* ORF into either pLgatewayIRESEYFP or pLgatewaySN to generate retroviral delivery vectors carrying either the EYFP or G418 resistance marker. Details of these different clones as well as the abbreviations used for their designation are summarized in Table 1.

Fv1 open reading frames from *M. m. macedonicus*, *M. n. minutoides*, *M. n. gratus*, *M. n. setulosis*, *M. n. triton* and *M. p. saxicolor* were synthesized chemically (GENEART, Life Technologies) based on their published sequences [40] with added attB sites and introduced into pLgatewayIRESEYFP via pDNR221. These clones are also summarized in Table 2.

### Construction of chimeric Fv1s

*Fv1* chimeras were generated by overlapping PCR. Briefly, a 5′ fragment was amplified from one parental sequence while a 3′ fragment was amplified from the other. The two fragments were then combined in a third amplification reaction using forward and reverse primers that annealed to the 5′ and 3′ ends of *Fv1* respectively. Internal primer pairs were designed to target regions of identity between the two parental sequences. The sequences of the primers are shown in Table S1.

To generate the Fv1nC series, the 5′ fragments were amplified from Fv1^n^ using TopoFv1F and either C1Rev, C3Rev, C4Rev or C5Rev, while the 3′ fragments were amplified from *Fv1caroli* (*CAR1*) using either C1F, C3F, C4F or C5F and Fv1caroliRev. The 2 fragments were joined in a reaction using TopoFv1F and Fv1caroliRev to yield Fv1nC1, Fv1nC3, Fv1nC4 and Fv1nC5. Similarly, the 5′ fragments for the reciprocal series Fv1Cn were amplified from *Fv1caroli* (*CAR1*), using the same primer pairs as the Fv1nC series, while the 3′ fragments were amplified from Fv1^n^ using either C1F, C3F, C4F or C5F and Fv1^n^Rev. These fragments were joined using primer pair TopoFv1F and Fv1nRev, yielding Fv1Cn1, Fv1Cn3, Fv1Cn4 and Fv1Cn5.

The 5′ fragments for the Fv1nS series were amplified from Fv1^n^ using TopoFv1F and either S1Rev, S2Rev or S3Rev, while the 3′ fragments were amplified from *Fv1spretus* (*SPR1*) using either S1F, S2F or S3F and Fv1spretusRev. The 2 fragments were joined together in a reaction using TopoFv1F and Fv1spretusRev to yield Fv1nS 1, Fv1nS2 and Fv1nS3. Similarly, the 5′ fragments for the reciprocal series Fv1Sn were amplified from *Fv1spretus* (*SPR1*), using the same primer pairs as the Fv1nS series, while the 3′ fragments were amplified from Fv1^n^ using either S1F, S2F or S3F and Fv1^n^Rev. These fragments were joined using primer pair TopoFv1F and Fv1^n^Rev, yielding Fv1Sn1, Fv1Sn2 and Fv1Sn3.

The chimeric fragments were cloned into pENTR/D-TOPO (Invitrogen) and verified by sequencing before transferring into the retroviral vector pLgatewayIRESEYFP.

### Site directed mutagenesis

The point mutants were generated by site directed mutagenesis using the primer pairs listed in Table S1. Mutagenesis was carried out in 50 microlitre reactions containing 2.5 units of Pfu ultra, 10 ng of template, 0.2 mM dNTP and 125 ng each of the forward and reverse primer. The reaction was performed in a thermal cycler at 95°C for 2 minutes followed by 18 cycles of 95°C for 30 seconds, 55°C for 1 minute and 68°C for 9 minutes 30 seconds. The PCR product was then digested with10 units of DpnI (Roche) for 1 hour before transforming XL10Gold cells (Agilent technologies). Colonies were screened by restriction digest and the mutations were verified by sequencing.

### Cells and virus production

MDTF and 293T cells were maintained in DMEM containing 10% foetal calf serum and 1% penicillin and streptomycin. Viruses were made by the transient transfection of 293T cells as previously described [19], [51]. Delivery viruses were produced by co-transfecting pcz-VSVG, pHIT60 and a retroviral vector containing Fv1 and either the EYFP or G418 resistance gene. N-, B- and NB-tropic MLV tester viruses were generated by co-transfection of pczVSVG, pczCFG2fEFPf and either pCIGN, pCIGB or pHIT60 respectively, while the NR-tropic viruses were made using a mutagenized form of pCIGN as previously described [16]. EIAV tester viruses were made using pczVSVG, pONY3.1 and pONY8.4ZCG or pONY4.1Z [66], while PFV, SFV and FFV were produced with pciSFV-1envwt and either pczDWP001, pcDWS001 or pcDWF003 respectively [29]. HIV-1 tester viruses were generated by co-transfecting pczVSVG with p8.91 and pCSGW. MLV and HIV-1 were frozen in aliquots at −80°C while EIAV and foamy viruses were freshly prepared for each experiment.

### Restriction assays

Restriction activity was routinely assayed using transient two colour FACS analyses as described previously [19], [51]. Briefly, *Fv1* was introduced into MDTF cells together with an EYFP marker in a retroviral delivery vector. Three days post-transduction, the cells were challenged with tester viruses carrying the EGFP markers. The cells were then subjected to FACS analyses three days later and the percentages of tester virus positive cells in EYFP (i.e. *Fv1*) - positive and - negative cells determined and compared. Ratios of less than 0.3 were taken as restriction while those that were greater than 0.7 were taken to represent no restriction. Numbers between 0.3 and 0.7 were taken to represent partial restriction.

Alternatively, single cell clones stably expressing restricting Fv1s were derived by transducing MDTF cells in 12 well plates with limiting dilutions of retroviral vectors carrying *Fv1* and a G418 resistance marker. The cells from each well were transferred to a 10 cm dish and G418 was added to a concentration of 1 mg/ml. Well-separated colonies were picked from the dishes when they appeared 7 to 10 days after antibiotic selection was started. Typically, 6 to 8 colonies were picked for each *Fv1* cell line, expanded and tested for restriction before being used for virus titration. To titrate tester viruses, MDTF cells and their derivatives were seeded in 12 well plates at a density of 5×10^4^ cells per well 24 hours prior to infection. Increasing amounts of viruses carrying the EGFP marker were then added to the wells and the percentage of infected cells was determined by FACS 3 days post infection.

### Quantitative PCR

MDTF cells and their derivatives stably expressing *Fv1* were seeded in 6 well plates at a density of 5×10^5^ cells per well 24 hours prior to infection. The cells were transduced at an m.o.i. of 1 with equal amounts of viral vectors that had been pre-treated with 10 units/ml of DNase (Promega) for 1 hour at room temperature. The cells were harvested 7 or 18 hours post-infection for quantification of late RT products and 2 LTR circles respectively. Total genomic DNA was extracted using the DNeasy blood and tissue kit (Qiagen) and 250 mg or 500 mg was used for quantitative PCR to detect late RT products and 2 LTR circles respectively. Primers and probes directed against EGFP [67] were used for quantifying late RT products from MLV and FFV while those directed against LacZ were used for EIAV. The retroviral vectors fEGFPf and pHIT111 were used as standards for EGFP and LacZ quantification respectively. Primers and probes for the detection of MLV 2 LTR circles have been described previously [68]. In order to detect EIAV and FFV 2 LTR circles, primers and probes that amplified and bound to a fragment spanning the 2LTRs were designed. For EIAV 2 LTR circle detection, EIAV2LTRCF (5′ACTCAGATTCTGCGGTCTGAG3′), EIAV2LTRCRev (5′ACCCCTCATAAAAACCCCAC3′) and EIAV2LTRCprobe (5′FAM-CTCAGTCCCTGTCTCTAGTTTGTCTGTTCG-Tamra3′) were used while FFV2LTRCF (5′CCAGAACTCACATGAGTGGTG3′), FFV2LTRCRev (5′CTCATCGTCACTAGATGGCAG3′) and FFV2LTRCprobe (5′FAM-GAAGGACTAACCTATCCCAGGTATAGGCCG3-Tamra') were used for the quantification of FFV 2LTR circles. The primer pairs were used to amplify fragments spanning the 2 LTRs from genomic DNA of EIAV or FFV infected cells. The fragments were cloned into pCR-BluntII-TOPO (Invitrogen) to be used as standards. Quantitative PCR was performed in 25 ml reactions using the ABsolute QPCR Rox mix from Abgene with 300 nM of each primer and 200 nM of probe. A programme of 50°C for 2 minutes, 95°C for 15 minutes followed by 40 cycles of 95°C for 15 s and 60°C for 1 minute was employed in the Applied Biosystems 7500 real time PCR system.

### Phylogenetic analysis

Trees were generated using the MegAlign programme from the DNASTAR Lasergene package. The distance values were calculated using the Kimura distance formula that takes into account the number of non-gap mismatches and silent substitutions.

## Supporting Information

Figure S1 Comparison of the Fv1 sequence from different mice. Predicted amino acid sequence encoded by the *Fv1* gene from 20 different sources, compared to the *Fv1^b^* allele found in C57BL mice. Source designations are as given in Table 1. Single letter amino acid code; . = identical to Fv1b; - = deletion; * = stop.
(PDF)

Figure S2 Western blot analysis of Mus dunni cells transduced with *Fv1*s from wild mice. MDTF cells were transduced with retroviral vectors carrying the *Fv1* gene from wild mice and the EYFP marker such that 50% of the cells were transduced, as determined by flow cytometry. A western blot analysis was performed 3 days post transduction using 25 µg of total protein from the cell extracts as previously described [48].
(PDF)

Table S1 Primer sequences. The sequences of the primers used in this study are listed.
(DOCX)
