# Supplementary material for: Evolution of the Retroviral Restriction Gene Fv1: Inhibition of Non-MLV Retroviruses
Source: PLoS Pathog. 2014 Mar 6;10(3):e1003968. doi: 10.1371/journal.ppat.1003968 (PMC3948346; doi:10.1371/journal.ppat.1003968)
Supplement: Figure S1 — Comparison of the Fv1 sequence from different mice. Predicted amino acid sequence encoded by the Fv1 gene from 20 different sources, compared to the Fv1b allele found in C57BL mice. Source designations are as given in Table 1. Single letter amino acid code; . = identical to Fv1b; - = deletion; * = stop. (PDF) [file ppat.1003968.s001.pdf]

```
       1          11         21         31         41         51         61         71         81         91         101        111
       |          |          |          |          |          |          |          |          |          |          |          |
C57BL  MNFPRALAGF SSWLFKPELA EDSPDNDSPD NDTVNPWREL LQKINVADLP DSSFSSGKEL NDSVYHTFEH FCKIRDYDAV GELLLAFLDK VTKERDQFRD EISQLRMHIN DLKASKCVLG
AKR    .......... .......... .......... .......... .......... .......... .......... .......... .......... .......... .......... ..........
DBA    .......... .......... .......... .......... .......... .......... .......... .......... .......... .......... .......... ..........
129    .......... .......... .......... .......... .......... .......... .......... .......... .......... .......... .......... ..........
LG/J   .......... .......... .......... .......... .......... .......... .......... .......... .......... .......... .......... ..........
MOL    .......... .......... .......... .......... .......... .......... .......... .......... .......... .......... .......... ..........
BAC    .......... .......... .......... .......... .......... .......... .......... .......... .......... .......... .......... ..........
CAS1   .......... .......... .......... .......... .......... .......... .......... .......... .......... .......... .......... ..........
CAS2   .......... .......... ...L...... .......... .......... .......... .......... .......... .......... .......... .......... ..........
SPR1   .......... .......... .......... ....S..... .......... .......... .....D.... .......... .......... .......... .......... ..........
SPR2   .......... .......... .......... ....S..... .......... .......... .....D.... .......... .......... .......... .......... ..........
MAC1   .......... .......... .......... ....S..... .......N.. .......... .....D.... .......... .......... .......... .......... ..........
SPI1   .......... .......... .......... -...S..... .......... .......... .....G.... .......... .......... .......... .......... ..........
CAR1   .......... .......... .......--- EN........ .......... .........V .....D.... .......... .......... .......... .......... ..........
CAR2   .......... .......... ..T....--- EN........ .R........ .......... .V...D.... .......... .......... .......... .......... ..........
CER    .......... .......... .......--- ENP....... .......... .......... .....D.... .......... .......... .......... ......L... ..........
DUN    .......... .......... .....K.--- EN........ .........L .......... .....D.... .......GQL ASCFWH.WI. *
COO    .......... .......... .......--- EN........ .......... .......... S....D.... .......... .......... .......... .......... ..........
FAM    .......... .......... .......--- EN........ .......... .......... .....D.... .......... .......... .......... .......... ..........
MIN    .......... .......... .....S.--- EN........ .....A.... ........K. .....D.... .......N.. .......... .......... .......... ..........
PLA    ...L...... .......D.. .....G.--- EN........ ....SA.... .......... ....CD.... .......... .......... .......... ........V. ..........

       121        131        141        151        161        171        181        191        201        211        221        231
       |          |          |          |          |          |          |          |          |          |          |          |
C57BL  ETLLSYRHRI EVGEKQTEAL IVRLADVQSQ VMCQPARKVS ADKVRALIGK EWDPVTWDGD VWEDIDSEGS EEAELPTVLA SPSLSEESGY ALSKERTQQD KADAPQIQSS TSLVTSEPVT
AKR    .......... .......... .......... .......... .......... .......... .......... .......... .......... .......... .......... ..........
DBA    .......... .......... .......... .......... .......... .......... .......... .......... .......... .......... .......... ..........
129    .......... .......... .......... .......... .......... .......... .......... .......... .......... .......... .......... ..........
LG/J   .......... .......... .......... .......... .......... .......... .......... .......... .......... .......... .......... ..........
MOL    .......... .......... .......... .......... .......... .......... .......... .......... .......... .......... .......... ..........
BAC    .......... .......... .......... .......... .......... .......... .......... .......... .......... .......... .......... ..........
CAS1   .......... .......... .......... .......... .......... .......... .......... .......... .......... ..F....... .......... ..........
CAS2   ..F....... .......... .........R .......... .......... .......... .......... .......... .......... .......... .......... ..........
SPR1   .......... ...K...... .......... .......... .......... .......... .........T .......... .......... .....L.... .......... ..........
SPR2   .......... ...K...... .......... .......... .......... .......... .........T .......... .......... .....L.... .......... ..........
MAC    .......... .......... .......... .......... .......... .......... .........T .......... .......... .......... .......... ..........
SPI    .......... .......... .......... .......... .......... .......... .........T .......... .......... .......... .......... ..........
CAR1   .......... .......... .......... .......... .......... .......... .........T ...ALLPTVLA......... ......A... ......N... ..........
CAR2   .......... .......... ......L... .......... .......... .......... .......D.T .....I.... .......... ......A... ......N... ..........
CER    .......... .......... ......L... .......... .......... .......... .......D.T .......... .......... .......... ...VS.N... ..........
DUN
COO    .......... .......... ......L... ......Q... .......... .......... .......D.T .......... .......... ......I*
FAM    .......... .......... ......L... .......... .......... D......... .......D.T .......... .......... .S........ ......K... ..........
MIN    .......... .......... ......L... .T........ .......... .......... .......D.T .K.....DS. ..P....... .....L.... ..EV..N... ..........
PLA    .......... .......... ......L... IT.......F .......... .......... .......D.. .K.....AS. .......... .......... ...VR.N... .....A....


       241        251        261        271        281        291        301        311        321        331        341        351
       |          |          |          |          |          |          |          |          |          |          |          |
C57BL  RPKSLSDLTS QKHRHTNHEL NSLAHSNRQK AKEHARKWIL RVWDNGGRLT ILDQIEFLSL GPLSLDSEFN VIARTVEDNG VKSLFDWLAE AWVQRWPTTR ELQSPDTLEW YSIEDGIERL
AKR    .......... .......... .......... .......... .......... .......... .......... .......... .......... .......... .......... .......K..
DBA    .......... .......... .........Q .......... .......... .......... .......... .......... .......... .......... .......... .......K..
129    .......... .......... .......... .......... .......... .......... .......... .......... .......... .......... .......... .F.....K..
LG/J   .......... .......... .........Q .......... .......... .......... .......... .......... .......... .......... .......... .F.....K..
MOL    .......... .......... .......... .......... .......... .......... .......... .......... .......... .......... .......... .F.....K..
BAC    .......... .......... .......... .......... .......... .......... .......... .......... .......... .......... .......... .......K..
CAS1   .......... .......... .......... .......... .......... .......... .......... .......... .......... .......... .......... .......K..
CAS2   .......... .......... .........Q .......... .......... .......... .......... .......... .......... .......... .......... .......K..
SPR1   .......... .......... K...Q..C.Q .......... .......... .......... .......... .......... .......... ........P. ...T...... .......K..
SPR2   .......... .......... K...Q..C.Q .......... .......... .......... .......... .......... .......... ........P. ...T...... .......K..
MAC    .......... .......... K...Q....Q .......... .......... ....N..... .......... .......... .......... .......... ...T...... .......K..
SPI    .......... ...G...... K...Q....Q .......... .......... ....N..... .......... .......... .......... .......... ...T...... .......K..
CAR1   ......G.I. .......... K...Q.Y... .......... .......... ....T..... .......... ......A... .......... ......TTTR...T..A.K. .Y.....K..
CAR2   .......... .......... K...Q..H.Q .......... .......... ....T..... .......... .......... .......... .......... ...T..A... ...G...K..
CER    .......... .......... Y...Q....Q E......... .......... ....T..... .......... ......A... .......... .......... ...M..A... .......K..
DUN
COO
FAM    .......... ......K... D...L....Q .......... .......... .......... .......... .......... .......... .......... ...T...... .......K..
MIN    .......... ...C..DQ.. K...Q..C.N .G.R.G.... .......... ..N.T..... .......... .......E.. .......... ........I. ...T...... FYV....K..
PLA    ....--.... .......Q.. ....QA...R ...R.T.... .......... ....T..... .......... .......E.. .......... .......... ...T...... ..V....K..

       361        371        381        391        401        411        421        431        441
       |          |          |          |          |          |          |          |          |
C57BL  RELGMIEWLC VKATCPQWRG PEDVPITRAM RITFVRETRE TWKSFVFSLL CIKDITVGSV AAQLHDLIEL SLKPTAAGLT SVGSVGVLSL SPWKHQSNS*
AKR    .......... .......... ..................V. .......... .......... .......... .......TKL *
DBA    .......... .......... .......... ........V. .......... .......... .......... .......TKL *
129    .......... .......... .......... ........V. .......... .......... .......... .......TKL *
LG/J   .......... .......... .......... ........V. .......... .......... .......... .......TKL *
MOL    .......... .......... .......... .......... .......... .......... .......... .......... .......... .........*
BAC    .......... .......... .......... ........V. .......... .......... .......... .......... .......... .........*
CAS1   .......... .......... .......... ........V. .......... .......... .......... .......... .......... .........*
CAS2   .......... .....S.... .......... ........V. .......... .......... .......... .......... AGLASVGSVG VLSLSPWKHQ SNS*
SPR1   ....I..... .......... .......... ........V. .......... .......... .......... .........A .......... .....*
SPR2   ....I..... .......... .......... ........V. .......... .......... .......... .........P AGLASVGSVG VLSLSPWKH*
MAC    .......... .......... ...A....G. .......... N......... .......... .......... .......... AGLASVGSVG VLSLSPWKH*
SPI    .......... .......... ...A...... .......... .......... .......... .......... .......... AGLASVGSVG VLSLSPWKH*
CAR1   .......... .......... ...A...... ....Q..... .......... ........G. .......... .......... AGSAPVGSVA PVGSVAVLSL SPWKHQSNS*
CAR2   .......... .......... ...A...... .......... .......... ........G. .......... .......... AGSAPVGSVA VLSLSPWKHQ SNS*
CER    .......... ...S...... ...A...... .......... .......... .......... .......... .......... AGLAPVGSVS VLSLSPWKHQ SNS*
DUN
COO
FAM    .......... .......... .......... ........V. .......... .......... .......... ....A...RG GARF*
MIN    .......... ...P..R... .D.A...... ..V.....E. S......... .......... ......IM.. RK.KP.GRGG ARF*
PLA    .......... .......... ...A...... ........I. D......... .......... .......... .KEASTREED F*
```

Figure S1

Comparison of the Fv1 sequence from different mice. Predicted amino acid sequence encoded by the *Fv1* gene from 20 different sources, compared to the *Fv1*^{b} allele found in C57BL mice. Source designations are as given in Table 1A. Single letter amino acid code; . = identical to Fv1b; - = deletion; * = stop.
