# Supplementary material for: Evolution of the Retroviral Restriction Gene Fv1: Inhibition of Non-MLV Retroviruses
Source: PLoS Pathog. 2014 Mar 6;10(3):e1003968. doi: 10.1371/journal.ppat.1003968 (PMC3948346; doi:10.1371/journal.ppat.1003968)
Supplement: Figure S2 — Western blot analysis of Mus dunni cells transduced with Fv1s from wild mice. MDTF cells were transduced with retroviral vectors carrying the Fv1 gene from wild mice and the EYFP marker such that 50% of the cells were transduced, as determined by flow cytometry. A western blot analysis was performed 3 days post transduction using 25 µg of total protein from the cell extracts as previously described [48]. (PDF) [file ppat.1003968.s002.pdf]

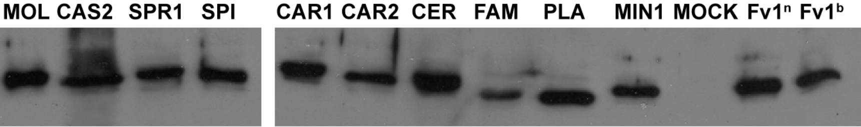
MOL
CAS2
SPR1
SPI
CAR1
CAR2
CER
FAM
PLA
MIN1
MOCK
Fv1n
Fv1b

Figure S2

Western blot analysis of Mus dunni cells transduced with *Fv1*s from wild mice. MDTF cells were transduced with retroviral vectors carrying the *Fv1* gene from wild mice and the EYFP marker such that 50% of the cells were transduced, as determined by flow cytometry. A western blot analysis was performed 3 days post transduction using 25 μg of total protein from the cell extracts as previously described (48).
