# Supplementary material for: Evolution of the Retroviral Restriction Gene Fv1: Inhibition of Non-MLV Retroviruses
Source: PLoS Pathog. 2014 Mar 6;10(3):e1003968. doi: 10.1371/journal.ppat.1003968 (PMC3948346; doi:10.1371/journal.ppat.1003968)
Supplement: Table S1 — Primer sequences. The sequences of the primers used in this study are listed. (DOCX) [file ppat.1003968.s003.docx]

**Table S1.** PCR primers

| PL80 | GAAGCGGAAGAAGTCTCTTG |
| --- | --- |
| GT17 | TGGATAGTCGACATCTATACTATCTTGGTGAG |
| GatewayFv1F | GGCTCCACCATGAATTTCCCACGTGCGCTTG |
| GatewayFv1Rev | TGGGTGGATTCACATAGGAAGGTAACCCACCTC |
| UniversalF | GGGGACAAGTTTGTACAAAAAAGCAGGCTCCACCATG |
| UniversalRev | GGGGACCACTTTGTACAAGAAAGCTGGGTGGATTCA |
| TopoFv1F | caccatgaatttcccacgtgcgc |
| Fv1^n^Rev | tcagagttttgtagctgctgttggctttaaac |
| Fv1caroliRev | TTAACTGTTGCTTTGATGTTTCCAGGG |
| Fv1spretusRev | TTAATGTTTCCAGGGAGAG |
| C1Rev | cactttcctcagacaaggatggagaggcc |
| C3Rev | cccttaaaatccatttcctagcatgttcc |
| C4Rev | catgcttcagccaaccaatcaaacaaactc |
| C5Rev | caagtagcttttacacaaagccactctatc |
| C1F | catccttgtctgaggaaagtggttatgcc |
| C3F | ggaaatggattttaagggtgtgggataatg |
| C4F | gattggttggctgaagcatgggtccagag |
| C5F | gctttgtgtaaaagctacttgtccacagtg |
| S1Rev | ggatggagaggccaagacagtgggcaactc |
| S2Rev | cttaaaatccatttcctagcatgttcctttgc |
| S3Rev | ctgtggacaagtagcttttacacaaagccac |
| S1F | ctgtcttggcctctccatccttgtctgagg |
| S2F | catgctaggaaatggattttaagggtgtggg |
| S3F | gtaaaagctacttgtccacagtggaggggc |
| Fv1caroli delta336 F | GAGATGGCCTACTACAAGAGAGCTGC |
| Fv1caroli delta336 Rev | GCAGCTCTCTTGTAGTAGGCCATCTC |
| Fv1caroli T343S F | CAAGAGAGCTGCAGtCGCCTGACGCCC |
| Fv1caroli T343S Rev | GGGCGTCAGGCGaCTGCAGCTCTCTTG |
| Fv1caroli A346T F | GCAGACGCCTGACaCCCTGAAGTGG |
| Fv1caroli A346T Rev | CCACTTCAGGGtGTCAGGCGTCTGC |
| Fv1caroli K348E F | GCCTGACGCCCTGgAGTGGTATTATATTG |
| Fv1caroli K348E Rev | CAATATAATACCACTcCAGGGCGTCAGGC |
| Fv1caroli Y351S F | CTGAAGTGGTATTcTATTGAGGATGGG |
| Fv1caroli Y351S Rev | CCCATCCTCAATAgAATACCACTTCAG |
| Fv1^n^ Ins 339T F | GAGATGGCCTACTactACAAGAGAGCTGC |
| Fv1^n^ Ins 339T Rev | GCAGCTCTCTTGTagtAGTAGGCCATCTC |
| Fv1^n^ S344T F | CAAGAGAGCTGCAGaCGCCTGACACCC |
| Fv1^n^ S344T Rev | GGGTGTCAGGCGtCTGCAGCTCTCTTG |
| Fv1^n^ T347A F | GCAGTCGCCTGACgCCCTGGAGTGG |
| Fv1^n^ T347A Rev | CCACTCCAGGGcGTCAGGCGACTGC |
| Fv1^n^ E349K F | GCCTGACACCCTGaAGTGGTATTCTATTG |
| Fv1^n^ E349K Rev | CAATAGAATACCACTtCAGGGTGTCAGGC |
| Fv1^n^ S352Y F | CTGGAGTGGTATTaTATTGAGGATGGG |
| Fv1^n^ S352Y Rev | CCCATCCTCAATAtAATACCACTCCAG |
| Fv1^n^ E349K S352Y F | GCCTGACACCCTGaAGTGGTATTaTATTG |
| Fv1^n^ E349K S352Y Rev | CAATAtAATACCACTtCAGGGTGTCAGGC |
| Fv1^n^ R216L F | gccttgtctaaagaacTcacccagcaggac |
| Fv1^n^ R216L Rev | gtcctgctgggtgAgttctttagacaaggc |
| Fv1^n^ N261K F | CTAAtCATGAACTCAAaTCACTTGCTCACTC |
| Fv1^n^ N261K Rev | GAGTGAGCAAGTGAtTTGAGTTCATGaTTAG |
| Fv1^n^ H265Q F | ctcaattcacttgctcaAtcaaatcgccaaaag |
| Fv1^n^ H265Q Rev | cttttggcgatttgaTtgagcaagtgaattgag |
| Fv1^n^ R268C F | cttgctcactcaaatTgccaaaaggcaaagg |
| Fv1^n^ R268C Rev | cctttgccttttggcAatttgagtgagcaag |
| Fv1^n^ K270Q F | cactcaaatcgccaaCaggcaaaggaacatg |
| Fv1^n^ K270Q Rev | catgttcctttgcctGttggcgatttgagtg |
| Fv1spretus L216R F | GCCTTGTCTAAAGAACgCACCCAGCAGGAC |
| Fv1spretus L216R Rev | GTCCTGCTGGGTGcGTTCTTTAGACAAGGC |
| Fv1spretus K261N F | CTAACCATGAACTCAAtTCACTTGCTCAATC |
| Fv1spretus K261N Rev | GATTGAGCAAGTGAaTTGAGTTCATGGTTAG |
| Fv1spretus Q265H F | CAAATCACTTGCTCAcTCAAATTGCCAACAG |
| Fv1spretus Q265H Rev | CTGTTGGCAATTTGAgTGAGCAAGTGATTTG |
| Fv1spretus C268R F | CTTGCTCAATCAAATcGCCAACAGGCAAAGG |
| Fv1spretus C268R Rev | CCTTTGCCTGTTGGCgATTTGATTGAGCAAG |
| Fv1spretus Q270K F | CAATCAAATTGCCAAaAGGCAAAGGAACATG |
| Fv1spretus Q270K Rev | CATGTTCCTTTGCCTtTTGGCAATTTGATTG |
